# mRNA Vaccine Development for Emerging Animal and Zoonotic Diseases

**DOI:** 10.3390/v14020401

**Published:** 2022-02-15

**Authors:** Ting Le, Chao Sun, Jitao Chang, Guijie Zhang, Xin Yin

**Affiliations:** 1 State Key Laboratory of Veterinary Biotechnology, Harbin Veterinary Research Institute, The Chinese Academy of Agricultural Sciences, Harbin 150069, China; LeZTing@163.com (T.L.); sunchao@caas.cn (C.S.); 2 Departments of Animal Science, School of Agriculture, Ningxia University, Yinchuan 750021, China

**Keywords:** mRNA vaccine, infectious disease, zoonoses, immune response, viruses

## Abstract

In the prevention and treatment of infectious diseases, mRNA vaccines hold great promise because of their low risk of insertional mutagenesis, high potency, accelerated development cycles, and potential for low-cost manufacture. In past years, several mRNA vaccines have entered clinical trials and have shown promise for offering solutions to combat emerging and re-emerging infectious diseases such as rabies, Zika, and influenza. Recently, the successful application of mRNA vaccines against COVID-19 has further validated the platform and opened the floodgates to mRNA vaccine’s potential in infectious disease prevention, especially in the veterinary field. In this review, we describe our current understanding of the mRNA vaccines and the technologies used for mRNA vaccine development. We also provide an overview of mRNA vaccines developed for animal infectious diseases and discuss directions and challenges for the future applications of this promising vaccine platform in the veterinary field.

## 1. Introduction

Vaccination has made a tremendous contribution to human and veterinary medicine [1]. Two major viral diseases, smallpox and rinderpest, have been eradicated worldwide with the help of vaccination [2,3]. During the COVID-19 pandemic, different types of vaccines have been developed and authorized for human use [4,5,6,7,8,9,10]. Among them, COVID-19 mRNA vaccines have shown notable effectiveness in disease prevention and have attracted public attention. This is the first time ever a mRNA vaccine has been licensed for human use. The emergence of mRNA vaccines has fundamentally revolutionized vaccine development [8,11,12,13,14].

**Figure 1:**
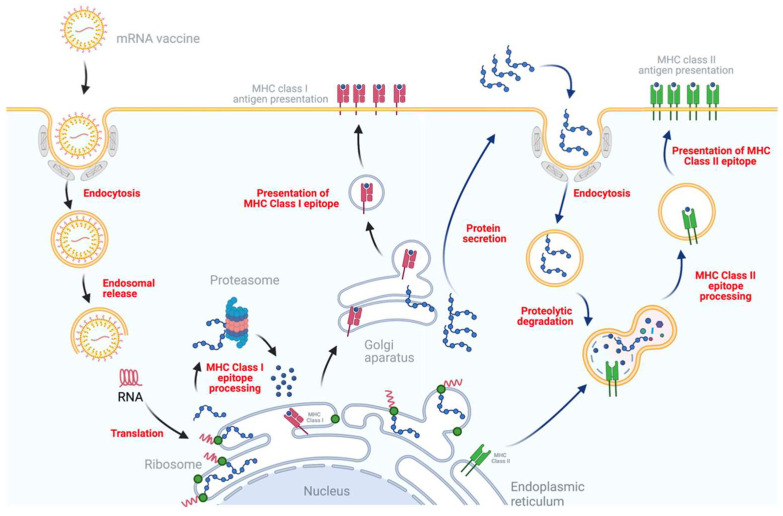
Antigen translation and presentation of mRNA vaccines in host cells. The mRNA encapsulated in the lipid carrier molecules transits to the cytosol via endocytosis, then mRNA is released by endosomal escape. After entering cytosol, cellular translation machinery is utilized to produce an antigen of interest. The ubiquitin-proteasome system then degrades intracellular antigen into peptides that can be presented by major histocompatibility complex (MHC) class I molecules. Lastly, MHC I -epitope complex is recognized by CD8^+^ T cells to trigger the specific immune responses. In addition, the antigen of interest secreted to the extracellular domain is ingested by antigen-presenting cells (APCs), such as dendritic cells, macrophages, and langerhans cells. Following proteolytic degradation and presentation by MHC class II molecules, the MHC II -epitope complex is recognized by CD4^+^ T cells to induce the CD4^+^ mediated immune responses.

In general, the mRNA encoding the immunogens is encapsulated in a lipid shell. Once delivered into the cytoplasm of target cells, the host cell machinery directs the translation of the antigen proteins that induce effective and long-lasting immune responses (Figure 1). Compared to conventional vaccines, mRNA vaccines have the potential for high potency, low-cost manufacture, rapid development, and safe delivery [14,15,16,17]. Additionally, the manufacture of mRNA vaccines does not require cell cultures or toxic chemicals, resulting in no chemical or biological contaminations in the final products. Furthermore, mRNA does not integrate into the host genome and so does not pose the risk of insertional mutagenesis and carcinogenesis. Lastly, scaled-up manufacturing to commercial levels at unprecedented scale and speed presents opportunities to effectively address emerging and re-emerging infectious diseases [18]. Therefore, mRNA vaccines represent a promising alternative to seed the field of vaccinology.

Animal infectious diseases remain a considerable challenge that impact animal health and food security [19]. In 2019, the spread of African swine fever virus (ASFV) in China killed nearly half of the pig population, leading to huge economic losses for the swine industry [20,21,22]. Foot-and-mouth disease (FMD), lumpy skin disease (LSD), porcine reproductive and respiratory syndrome (PRRS), African horse sickness (AHS), etc., also cause frequent outbreaks worldwide [23,24,25,26,27]. Notably, nearly two-thirds of the pathogens affecting humans originated from animals, such as the avian influenza virus, rabies virus, hepatitis e virus (HEV), and the recently emerged coronavirus called SARS-CoV-2. Prevention by vaccination is considered the most successful intervention strategy against animal infectious diseases, particularly zoonoses [28]. Currently, three main types of vaccines, including inactivated vaccines, live attenuated vaccines and recombinant subunit vaccines, are widely used in the veterinary field. However, these conventional vaccines present numerous disadvantages, particularly related to safety and efficacy concerns, such as reversion to virulence for live attenuated vaccines, poor immunogenicity for inactivated vaccines, and limited protective immunity against a heterogeneous virus for recombinant subunit vaccines (Table 1) [29].

Generally, the characteristics of an ideal vaccine for animal infectious diseases include: (1) broad-spectrum protection against all isolates in all the infected species to limit the potential transmission; (2) possibility of discrimination between infected and vaccinated animals; (3) no recombination between vaccines and field strains; (4) strong and long-lasting immune responses; (5) inexpensive manufacturing and simple administration. Therefore, novel vaccine formats are urgently needed. Here, we systematically reviewed recent advances in mRNA vaccine development and provided new insights for advancing mRNA vaccines in the veterinary field.

## 2. Forms of mRNA Vaccines

### 2.1. Non-Amplifying mRNA Vaccines

The classification of mRNA vaccines includes two major types: non-amplifying RNA vaccine and self-amplifying RNA (saRNA) vaccine [30]. Like natural mRNA, the fundamental structure of non-amplifying mRNA consists of a 5′ cap, 5′ untranslated region (UTR), the gene of interest encoding region, 3′ UTR, and the Poly(A) tails.

A 5′ cap structure is an essential invariant for efficient protein production by stabilizing the mRNA molecule. Addition of the 5′cap can be achieved either via a post-transcriptional enzymatic reaction guided by the vaccinia virus-capping enzyme or a co-transcriptional reaction by incorporation of the synthetic cap or anti-reverse analogues. Additional post-translational modifications such as 2′-O-methylation to introduce a cap 1 structure into an in vitro transcribed mRNA were proved to further improve the translational efficiency and to prevent undesirable immune responses [31,32,33].

The 5′ and 3′ UTR elements flanking the coding sequence also play an important regulatory role in the stability and translation of mRNA. A wide range of 5′ and 3′ UTRs originated from viral or eukaryotic genes have been deployed to promote mRNA in-cell stability and translation efficiency [34]. It appears that the composition and structure of the UTRs are the key determinants of the intracellular stability of mRNA. Among them, the 5′ and 3′ UTR from human hemoglobin subunit beta (hHBB), one of the most efficiently expressed mammalian mRNAs, was shown to confer higher stability and higher potential for protein production [34]. Of note, the UTRs’ combination for high protein expression is likely to be cell-type and species-dependent. Thus, to achieve superior translational performance, the optimal UTRs need to be selected from naturally occurring UTRs.

The poly(A) tails additionally have an important impact on protein translation by protecting the transcriptional body from exonucleolytic degradation. The length of the poly(A) tail seems to be highly associated with the translation efficiency in a cell-type dependent manner [35,36,37]. At present, most therapeutic mRNAs bear a poly(A) tail with a length of approximately 50~100 nucleotides. The tail can be incorporated into the mRNA transcripts either by poly(A) polymerase-mediated tailing or by direct synthesis of poly(A) tails using the plasmid template harboring poly(T) sequences during the in vitro transcription reaction.

In addition to the inclusion of regulatory elements in the non-amplifying RNA molecules, modification of the gene coding regions has been widely used to increase protein production from mRNA. Common strategies include chemical alterations of mRNA molecules with N1-methylpseudouridine, pseudouridine or other nucleoside analogues, substitution of rare codons with synonymous codons, and enrichment of GC content in the mRNA molecules. It has been shown that the replacement of uridine with pseudouridine confers protection from mRNA degradation mediated by RNase L [38]. Moreover, the innate immune response is significantly attenuated in vivo with this modified mRNA, compared to non-modified mRNA [39,40,41]. Thus, both the Moderna and Pfizer–BioNTech SARS-CoV-2 vaccines were designed to incorporate pseudouridine in the mRNA molecules [8,13].

Of note, during in vitro transcription, numbers of contaminants such as prematurely terminated transcripts or partially degraded mRNA molecules might be generated. The purification of mRNA by high performance liquid chromatography (HPLC) could be utilized to partially eliminate the expression of interferons by removing dsRNA contaminants. Therefore, removal of these contaminants via chromatographic techniques is required to produce purified mRNA with superior translation capacity [39]. Overall, these innovations have overcome significant manufacturing bottlenecks in scalable mRNA synthesis processes.

### 2.2. Self-Amplifying mRNA Vaccines

A self-amplifying RNA (saRNA) vaccine structurally resembles the non-amplifying RNA vaccine. However, unlike the non-amplifying mRNA vaccine that only encodes genes of interest for antigen expression, saRNA-based vaccine contains additional genes encoding the viral RNA replication machinery for extended duration and magnitude of antigen production even with a minimal dose of RNA required [42]. In principle, various viral RNA backbones could be used as the backbone of saRNA-based vaccines. Here we will focus on alphaviruses such as the Venezuelan equine encephalitis virus (VEEV), Semliki Forest virus (SFV) or Sindbis virus; we will focus on those in this section since much work has been done with them [42,43,44].

Upon delivery of saRNA into the cytosol, non-structural proteins are initially translated from the incoming mRNA to assemble a multi-enzyme replication complex. A full-length negative-stranded RNA is then transcribed from the input mRNA in the formed replication factories. This newly synthesized negative-stranded RNA serves as a template to produce both full-length genomic mRNA and a shorter sub-genomic mRNA. The full-length genomic RNA mediates expression of more replicases for auto-replication, while the sub-genomic RNA transcribed at extremely high levels encodes the antigen protein to elicit substantially strong immune responses in the vaccinated hosts. As expected, duration of expression from self-amplifying mRNA is dramatically enhanced.

**Figure 2:**
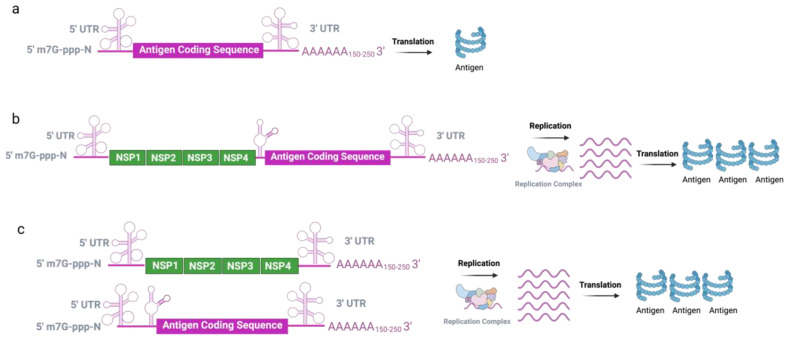
The types of mRNA vaccines. (**a**) The non-amplifying mRNA consists of 5′ cap, 5′ UTR, the gene of interest encoding region, 3′ UTR, and poly(A) tails; (**b**) The linear saRNA contains the genes of 5′ cap, 5′ UTR, intact RNA replication machinery, the gene of interest encoding region, and 3′ UTR; (**c**) The trans-amplifying system harbors an RNA-encoding RNA-replication machinery and another RNA-encoding antigen of interest.

Compared to non-amplifying RNAs, saRNA transcripts are much longer and have a high degree of secondary structure, which limits scaled-up production. To permit shorter lengths of RNA and to increase the stability, a trans-amplifying system based on a bipartite RNA vector system was then developed [45,46]. In this system, one vector cassette only harboring the replicase-encoding gene provides *in trans* alphaviral replicase machinery, while the second molecule originates from saRNA with the replicase deletion to express the vaccine antigen. With this developed two-vector system, nanogram doses of influenza hemagglutinin (HA)-encoding mRNA were sufficient to induce robust and protective neutralizing antibody responses against influenza in mice. In addition, the two-vector system showed a less-pronounced interference with cellular translation, leading to high amounts of antigen expression [45]. Therefore, the bipartite vector system may be advantageous over the single vector system regarding versatility and manufacturing. The structure of the main forms of mRNA vaccines is summarized in Figure 2.

An obstacle to full exploitation of saRNA-based vaccines is the fact that saRNA could induce aberrant innate host immune responses. During saRNA replication, double-stranded RNA (dsRNA) intermediates are highly produced, which can be potentially sensed by pattern recognition receptors such as melanoma differentiation-associated protein 5 (MDA-5), retinoic-acid-inducible gene I(RIG-I) and toll-like receptor 3 (TLR-3) [47,48]. These signaling transductions initiate the activation of interferon (IFN) and protein kinase R(PKR) signaling, leading to mRNA degradation and translation inhibition. As a result, the translational efficiency and duration of the vaccine antigen are limited. Thus, the potency of saRNA-based vaccine can be achieved by minimizing the IFN response. Recently, by using an in vitro evolution strategy through long-term culture of Jurkat cells transfected VEE replicon RNA, six mutations in the non-structural proteins were discovered to be associated with enhanced duration and antigen expression [49]. However, the underlying mechanisms by which the mutants escape from the IFN response for persistent replication remain unclear. In addition, co-delivery of the non-replicating mRNA encoding IFN antagonists such as vaccina virus (VACV) immune evasion protein E3 showed enhanced expression of the saRNA-encoded antigen through the suppression of PKR and IFN signaling activation [50,51]. Despite these advances, novel strategies to further mitigate the innate immune responses associated with saRNA vaccines are needed.

## 3. Delivery Platform and Formulation of mRNA Vaccines

Delivery systems and formulation of mRNA vaccines are key determinants of the magnitude and duration of vaccine antigen expression as well as the potency of the protective immune responses. Owing to the physicochemical properties of mRNA molecules, such as the large size and dense negative charge, naked mRNA hardly passes through the cell membrane. In addition, as an exogenous nucleic acid, the naked mRNA can be easily recognized by the pattern recognition receptors, leading to IFN responses and mRNA degradation, as described above. Therefore, the delivery vehicle is required to promote cellular uptake of mRNA and to increase their resistance to nuclease degradation. In recent years, great progress has been made in the field. The well-studied delivery tools for mRNA administration include ex vivo loading of dendritic cells, physical delivery via gene gun or electroporation [52], polymer-based delivery [53], and lipid nanoparticles (LNPs)-based delivery [54,55,56], among which LNPs have clearly emerged as one of the most appealing and widely used delivery tools. We will discuss the most recent progress on LNPs in detail below.

**Figure 3:**
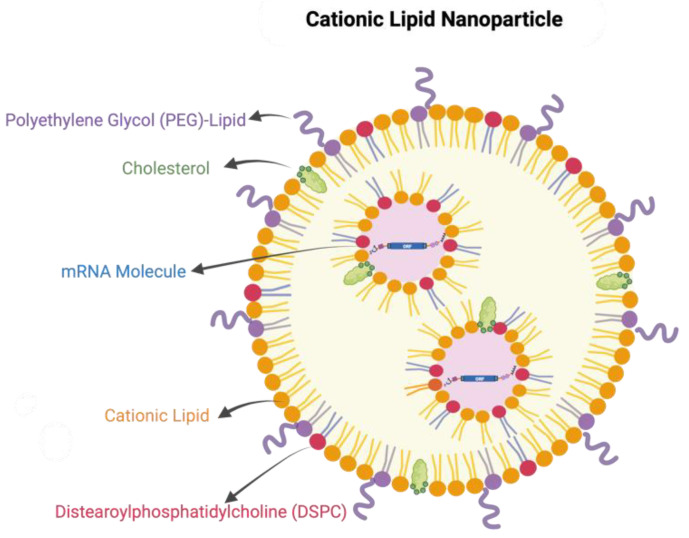
The structure of the cationic lipid nanoparticle. Cationic lipid nanoparticle mainly consists of cationic lipid, distearoylphosphatidylcholine (DSPC), lipid-linked polyethylene glycol (PEG), cholesterol, and mRNA molecules. Cationic lipids interact with the negatively charged mRNA to form the particles. DSPC enhances particle stability, delivery efficacy, and biodistribution. Cholesterol can improve LNP stability by modulating membrane integrity and rigidity. PEG-lipids can further improve LNP stability and control particle size.

LNPs usually contain one or more functional components that are essential for the delivery of mRNA. The three formulation components include: (1) an ionizable or cationic lipid material such as 1,2-di-O-octadecenyl-3-trimethylammonium propane (DOTMA), N1,N3,N5-tris (3-(didodecylamino)propyl) benzene-1,3,5-tricarboxamide (TT3), and N,N-Dimethyl-2,3-bis[(9Z,12Z)-octadeca-9,12-dienyloxy]propan-1-amine (DLinDMA) that mediate the encapsulation of the negatively charged mRNA molecules via electrostatic interactions; (2) lipid-linked polyethylene glycol (PEG) and cholesterol that could stabilize the nanoparticles during the preparation and increase the half-life of formulations after in vivo administration; and (3) phospholipids that participate in the formation of the lipid bilayer structure (Figure 3). The lipid nanoparticles of two approved COVID-19 mRNA vaccines contain an ionizable lipid, a PEGylated lipid, cholesterol, and the phospholipid distearoylphosphatidylcholine (DSPC). The molar ratios of the cationic lipid; PEG-lipid; cholesterol; and DSPC are (46.3:1.6:42.7:9.4) for Comirnaty (the Pfizer vaccine) and (50:1.5:38.5:10) for Spikevax (the Moderna vaccine) [8,13]. Those nanoparticles contain approximately 100 mRNA molecules per lipid nanoparticle with a diameter of 80–100 nm.

To efficiently assemble an LNP-mRNA complex, a wide variety of formulation methods have been developed [57,58,59]. Compared with the conventional methods such as direct mixing, film hydration, and reverse phase evaporation, microfluidic hydrodynamic focusing represents the most advanced platform for in vivo use owing to its higher reproducibility and stability [60,61]. The RNA in the aqueous phase and the lipids in the solvent phase can be rapidly diffused in the central channel of a continuous-flow microfluidic device. During the controlled, rapid mixing of these two phases from laminar flow, positively charged ionizable lipids or cationic lipids interact with the negatively charged mRNA to form the micelles and particles with high encapsulation rates [62]. Although the resulting lipid nanoparticles exhibit relatively homogeneous appearances, continuing research into diverse platforms for efficient formulation is still a priority to guide the development of efficient intracellular delivery systems.

In the past years, a plethora of studies have shown that the use of LNP technology could dramatically facilitate the delivery of mRNA and enhance antigen expression [63]. A number of LNP-formulated drugs and vaccines have been approved for use in medical practice. LNP-encapsulated mRNA vaccines against HIV-1, influenza and Zika have been shown to elicit potent antigen-specific CD4^+^ and CD8^+^ T cell responses as well as neutralize antibodies in the relevant animal models [64,65,66]. In addition to protecting mRNA from degradation and promoting cellular uptake, the LNP formulation possesses the intrinsic adjuvant activity that could mediate induction of strong T follicular helper (Tfh) cell, germinal center B cell, long-lived plasma cell and memory B cell responses in mice independently of mRNA [67,68]. Notably, the presence of the ionizable lipid component and the induction of the pro-Tfh cytokine IL-6 are essential for this adjuvant activity [68]. Altogether, these studies highlight the importance of LNPs for effective mRNA vaccines, and more efforts in understanding the mechanism of action are required in the future.

## 4. Mechanism of Immune Response Induced by mRNA Vaccines

**Figure 4:**
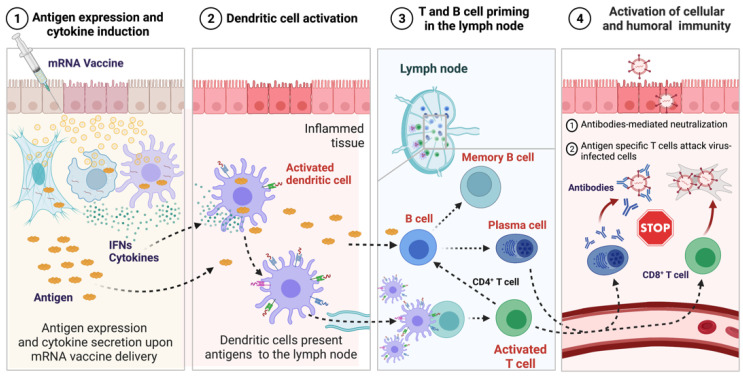
The innate and adaptive immune responses induced by mRNA vaccines. **①** Antigen expression and cytokine induction: With the administration of mRNA vaccine, mRNA can be recognized by RNA sensors, then the innate immune response is activated, resulting in production of cytokines and IFNs. **②** Dendritic cell activation: Dendritic cells are induced to maturation by produced IFNs and cytokines, and migrate to lymph for presenting the antigen of interest to T cells. **③** T and B cell priming in the lymph node: T cells are activated by the dendritic cell. The activated CD4+ helper T cells perform the assisted tasks, including promoting the maturation of plasma cells and secretion of antibodies. B cells induced by antigens differentiate into memory B cells and plasma cells with the help of CD4+ helper T cells and cytokines. **④** Activation of cellular and humoral immunity: The CD8+ cytotoxic T cells and antibodies transport to inflamed or infected tissue to clear the infection. The antigens can be cleared by antibodies-mediated neutralization and the death of virus-infected cells mediated by CD8+ cytotoxic T cells. The immune memory will be activated once infected with the same virus.

Administration of mRNA vaccines leads to the activation of both innate and adaptive immune systems (Figure 4), while the underlying mechanism remains to be elucidated. It is believed that mRNA stimulates innate immunity through two kinds of RNA sensors: endosomal TLRs localized in the endosomal compartment and the RLRs in the cytoplasm [69,70]. The resulting signaling transduction leads to increased production of IFNs and cytokines, which in turn mediates the recruitment of immune cells such as macrophages, dendritic cells (DCs) and natural killer (NK) cells to shape the adaptive immune system. It is worth noting that mRNA sensing by the innate immune system also negatively affects antigen expression, thereby working against the vaccine’s effectiveness. As reported previously, humoral and T cell responses to mRNA vaccination were significantly enhanced in IFNAR1 and IFNAR 2 knockout mice or by co-administration of IFN antagonist [71,72]. Moreover, the inflammatory effects of type I IFNs were considered as the cause of systemic side effects upon the administrations of mRNA vaccines [73,74]. Therefore, strategies to avoid excessive innate immune responses such as modification of mRNA with pseudouridines should be prioritized in the development of succussive mRNA vaccines.

The administration route apparently influences the immune responses to mRNA vaccines as well. Compared with intramuscular (IM) injection, intradermal (ID) injection of mRNA vaccine resulted in stronger initial responses by activating local DCs [75,76]. Intravenous (IV) injection could directly expose the mRNA vaccines to immune cells circulating in the blood. It may enhance the activation efficacy, but introduce systemic side effects such as spleen injury and lymphocyte depletion due to the induction of cytokine storm and recruitment of cytotoxic T cells in organs containing antigens [76]. In summary, a better elucidation of the underlying immune mechanism for mRNA vaccines will promote the development of an effective mRNA vaccine. Further research is needed to determine which pathways of immune signaling are involved in mRNA vaccine immunizations.

## 5. Development of mRNA Vaccines for Prevention of Animal Infectious Diseases and Zoonoses

Currently, most of the mRNA vaccines were developed for protecting against zoonotic diseases such as Ebola, influenza, rabies, and Zika virus disease. Only limited mRNA vaccines have been developed in the veterinary field.

### 5.1. mRNA Vaccines for Foot-and-Mouth Disease

FMD remains one of the most contagious diseases of cloven-hoofed animals caused by foot-and-mouth disease virus (FMDV) in low and middle-income countries [77]. FMDV has been classified into seven serotypes (A, O, C, Asia1, SAT1, SAT2 and SAT3) based on the antigenic and genetic attributes. Due to the high antigenic and genetic variability, the vaccines matching to the dominant antigenic variants need to be updated in a timely fashion. Currently, most of the commercialized vaccines against FMD are chemically inactivated virulent strains, which have many shortcomings, including the high risk of virulent FMDV escaping from manufacturing facilities, poor immunogenicity, difficult serological differentiation between infected and vaccinated animals, and thermal instability [78].

Many attempts to develop safer next-generation FMD vaccines have been extensively launched in the past decade. As early as the 2010s, a study showed that immunization with the full-length genetically engineered FMDV mRNA could induce strong immune responses to FMDV in a mice model, providing the possibility that RNA-based vaccines can be developed in the natural host [79]. Since the full-length viral genome is infectious in the natural host, a subsequent study conducted by the same group investigated the antiviral effects of several synthetic non-infectious RNA molecules in mice challenged with FMDV. Interestingly, injection of the transcripts corresponding to the internal ribosome entry site (IRES) also provided a wide-range protection against viral infection in a serotype-independent manner [80]. Given that no neutralizing antibodies were produced in the immunized mice, the innate immune response induced by IRES RNA likely conferred protections against FMDV infection. Consistently, the immunomodulatory effect of those synthetic non-infectious RNA molecules was further proven as the titers of specific anti-FMDV antibodies were significantly increased in the mice co-administrated with FMDV vaccine and RNA molecules [81].

Altogether, the available data provided a promising path for the development of mRNA-based FMD vaccines, and new and more rational approaches are still needed. Recently, lines of evidence showed that the empty capsid vaccine generated by the co-expression of the FMDV P1-2A capsid precursor plus the 3C^pro^ via the baculovirus or vaccinia virus expression systems was effective against FMD in the natural host [82,83]. Therefore, with the significant progress made in COVID-19 mRNA vaccines, a synthetic thermo-stable RNA vaccine harboring the FMDV P1-2A plus 3C^pro^ encoding genes can be developed to protect livestock against emerging FMD strains. In addition, compared with traditional vaccines, the mRNA vaccine will hold the promise of overcoming limitations such as the need of an infectious virus or cell culture.

### 5.2. mRNA Vaccines for Rabies Disease

Rabies is the most lethal zoonotic disease caused by the rabies virus, belonging to the Lyssavirus family. Each year, rabies virus infection causes approximately 60,000 human deaths worldwide, and more than 95 percent of these cases are caused by animal bites, particularly by dog bites.

Rabies vaccines were initially produced in animal neural tissues by Louis Pasteur to control rabies [84]. To overcome the severe side effects caused by the myelin contaminant in inactivated viral vaccines, safer and efficacious vaccines were then developed using egg-culture based systems or cell-culture based systems. However, respective specific precautions are required to handle the infectious rabies virus, rendering the manufacturing slow and extremely expensive [85]. Thus, the development of safe, effective, and affordable vaccines for use in humans and animals remains important.

The first rabies mRNA vaccine candidate (CV7201) consists of a temperature-stable mRNA encoding the rabies virus glycoprotein (G) antigen and cationic protein protamine as stabilizer [86]. The administration of mRNA by intradermal injection in BALB/c mice induced the production of virus-neutralizing antibodies, which conferred protection against rabies virus infection [87]. The magnitude and breadth of neutralizing antibody responses in mice immunized with rabies mRNA vaccine were comparable to that in mice immunized with inactivated rabies vaccines. Notably, compared to the licensed vaccine, rabies mRNA vaccine induced a better cellular immune response, which was essential for protection. The protective humoral immune response was also observed in the domestic pig [88]. Importantly, the lyophilized rabies mRNA vaccine retained immunogenicity even stored at temperatures up to +40 °C. The phase 1 clinical trial further confirmed that rabies mRNA vaccine (CV7201) formulated with protamine was immunogenic in most participants when delivered with a needle-free injector. However, a high dose of mRNA (80–640 ug) was required for protection [86]. To reduce the dose, a novel formulation containing the same mRNA antigen as CV7201 encapsulated in LNPs has been assessed in a phase 1 clinical study. As reported recently, two low doses of LNP-encapsulated mRNA (1 ug or 2 ug) were sufficient to elicit the neutralizing antibody responses that met WHO criteria in all participants [89].

In addition to non-amplifying rabies mRNA vaccine, several saRNA-based rabies vaccines encoding the glycoprotein G antigen were developed. Intramuscular injection of LNP-encapsulated saRNAs at a low dose could induce a protective immune response with limited side effects, emphasizing the potential to prevent rabies in canine populations [90]. Despite these advances, none of these mRNA vaccines have been tested in dogs or cats. Therefore, orally administered mRNA vaccines can be considered fordevelopment for elimination of animal rabies in the future.

### 5.3. mRNA Vaccines for Influenza

Influenza is a highly contagious respiratory illness caused by influenza viruses that circulates in a wide range of animal species, including birds, pigs, and dogs. In addition to seasonal epidemics, four major pandemics were caused by influenza variants including H1N1 in 1918, H2N2 in 1957, H3N2 in 1968, and H1N1 again in 2009 [91,92].

Due to the antigenic drift and shift associated with influenza viruses, annual changes in the composition of influenza vaccines are required to achieve protection through vaccination. The current influenza virus vaccines used in humans or birds are inactivated, or live-attenuated vaccines produced in egg-, cell-, and protein-based systems. The process from designing to manufacturing typically takes 6–8 months. More importantly, influenza A virus can be transmitted from birds or pigs to humans, causing severe illness. However, rapid vaccine production following the emergence of an outbreak with the conventional approach is almost impossible.

Strategies utilizing the mRNA vaccine against the influenza virus have been extensively developed and studied in the past years. Immunization with the saRNA vaccine expressing hemagglutinin (HA) antigen from several different serotypes produced protection from a lethal homologous viral challenge in mice [93]. More importantly, two doses of mRNA-lipid nanoparticles encoding HA antigens induced much stronger T- and B-cell immune responses than the licensed trivalent inactivated influenza vaccine. Consistently, the protection against homologous and heterosubtypic viral infection was also induced in mice immunized with saRNA vaccine encoding nucleoprotein (NP) and Matrix protein (M1) by eliciting a strong antigen-specific T-cell response [94]. Chitosan-nanoparticle delivery of saRNA harboring viral HA and NP gene into dendritic cells (DCs) was reported to provide protective immunogenicity with acceptable tolerability profiles by inducing both B- and T-cell immune responses in mice [95]. An important consideration for influenza vaccines is how antigenic drift and shift could render available vaccine ineffective.

The mRNA vaccine platform can flexibly accommodate multiple antigens. As expected, multivalent mRNA vaccines containing the conserved antigens within HA stalk, NA, M2, and NP have been developed for providing broad-spectrum immune protection against almost all strains of influenza virus [96]. Recently, with the success of COVID-19 mRNA vaccines, Moderna’s quadrivalent mRNA vaccine (mRNA-1010) for seasonal influenza has entered phase 1 and 2 clinical trials, and the safety and immunogenicity of 3 dose levels of mRNA-1010 will be evaluated in the participants [97].

Moreover, considering that avian or swine influenza viruses can frequently jump to humans, causing severe diseases, vaccination of the natural hosts with mRNA vaccines matching the emerging virus strains may hopefully control new outbreaks from zoonotic transmission, as the rapid development and mass production of mRNA vaccines are feasible.

### 5.4. mRNA Vaccines for Zoonotic Mosquito-Borne Flaviviruses

Members of Flaviviridae family including Zika virus (ZIKV), Japanese encephalitis virus (JEV), and deer Powassan virus (POWV), predominately spread by arthropod vectors. Traditional vaccines developed by using chemically inactivated viruses or live-attenuated strains have successfully controlled the spread of yellow fever virus (YFV) [98]. Numerous vaccines, including these traditional vaccines, immunogenic recombinant subunit-based vaccines, DNA vaccines, mRNA vaccines and adenovirus vectors-based vaccines, have also been developed and have shown promise against other flaviviruses.

In 1998, RNA encoding of an attenuated strain of tick-borne encephalitis virus (TBEV) was developed as one of the first mRNA vaccines in infectious diseases [99]. Antibody responses were detected in the mice administered the attenuated RNA coated in gold particles. A similar study using a full-length TBEV mRNA incapable of producing infectious particles found that the mRNA-immunized mice can develop protective immunity against the lethal virus infection [100]. Additionally, the antibody titers in mRNA-immunized mice were comparable to those in mice immunized with inactivated virus, but the CD8^+^ T cell response was superior in mice immunized with mRNA [101]. In 2001, a SFV-based mRNA vaccine encoding the premembrane (prM) plus envelope (ENV) protein of louping-ill virus (LIV) was developed. Administration of 10 ug of naked mRNA via intramuscular injection provided partial protection from a lethal challenge of LIV in mice, indicating that the prM and ENV proteins are suitable targets for constructing vaccine candidates [102].

Since then, most of the mRNA vaccines against other flaviviruses have been designed by using prM and ENV proteins as major antigens. A LNP-encapsulated mRNA vaccine coding for prM/ENV proteins of ZIKV with 1-methyl pseudouridine modification was demonstrated to completely protect both immunocompetent and immunocompromised mice from a lethal challenge [67,103]. Additionally, no antibody-dependent enhancement (ADE) was observed in immunized mice upon infection. Compared to inactivated ZIKV vaccines, the magnitude of antigen specific CD8^+^ and CD4^+^ T cell responses was much greater, highlighting the importance of T cell responses in controlling ZIKV infection. The data from a phase I/II clinical trial showed that the ZIKA mRNA-1893 vaccine harboring the sequence that encodes for the structural proteins prME induced over 90% seroconversion upon a prime-boost vaccination with 10 ug doses [104].

ADE is a general concern for the development of effective dengue virus (DENV) vaccines. The immune complexes caused by non-neutralizing or sub-neutralizing antibodies can result in aggravated infection of other DENV serotypes and enhanced inflammatory responses [105]. A T cell-based mRNA vaccine targeting immunodominant T cell epitopes that generates a potent virus-specific T cell response was then developed to avoid ADE effects. A potent virus-specific CD8^+^ T cell response against DENV infection was induced to suppress virus infection upon vaccination [105,106]. Moreover, as the T cell epitopes in the non-structural proteins are conserved across different DENV serotypes, the induced T cell responses partially conferred the protections against all four serotypes.

Powassan virus (POWV), belonging to Flaviviridae family that is transmitted by the deer tick, has recently come to the public’s attention. A lipid LNP-encapsulated modified mRNA vaccine encoding prM and E protein of POWV induced a robust humoral immune response against POWV strains [107]. Altogether, these studies have spurred excitement for preventing zoonotic mosquito-borne flaviviruses using the mRNA vaccine platform.

## 6. The Future Potential and Challenges of mRNA Vaccines in the Veterinary Field

Although only few mRNA vaccines have been specifically studied in protecting against animal infectious diseases in their natural hosts, the success of mRNA vaccines in humans has paved the way for advancement in veterinary medicine. Virus infections remain the major perceived threats to the global health and industrial livestock production. The major viruses from poultry and livestock lacking effective strategies to control include ASFV, porcine reproductive and respiratory syndrome virus (PRRSV), porcine epidemic diarrhea virus (PEDV), FMDV, bovine viral diarrhea virus (BVDV), lumpy skin disease virus (LSDV), bovine leukemia virus (BLV), peste des petits ruminants virus (PPRV), and so on. The availability of an mRNA-based vaccines platform might strategically advance safe and effective vaccines to market for preventing these diseases.

### 6.1. T-Cell-Directed mRNA Vaccine for ASFV

T-cells perform a central role in cell-mediated immune response through directly destroying virus-infected cells upon recognition of viral epitopes [108]. Thus, T-cell-directed vaccines composed of the immunodominant T-cell epitopes from pathogens are supposed to potentially provide immune memory capable of stemming the reinfection.

African swine fever (ASF) caused by ASFV is a devastating disease in pigs characterized by high mortality and fatal hemorrhages. Due to the lack of effective vaccines, ASF has spread to many countries, including China, the world’s largest pork producer, resulting in the death of nearly seven million pigs in 2019 [20,109]. However, multiple live attenuated ASFV vaccine candidates with deletion of the virulence genes have been developed and have been shown to effectively protect against lethal ASFV infection. The large-scale production of these attenuated vaccines heavily relies on the acquirement of primary porcine alveolar macrophages or bone marrow cells, which is time and cost consuming [110,111,112]. Recently, highly conserved cytotoxic T-cell epitopes have been identified in ASFV encoded proteins through a computational vaccine design platform named iVAX [113,114]. A T-cell-directed ASF DNA vaccine containing the predicted swine MHC class I and class II epitopes was then developed, and the immunogenicity of this novel vaccine is being assessed [115,116,117]. Given the potential risk of integration into the host genome associated with DNA vaccines, the mRNA-based vaccine approach has the potential to lead to improvements for safe and effective ASF vaccines for marketing. Notably, the ASFV double-stranded DNA genome is approximately 170–194 kilobase pairs and codes for 150–170 open reading frames, of which almost half lack any known or predictable functions. Further investigation may identify more potential T-cell epitope(s) to facilitate developing a T-cell-directed mRNA vaccine with greater epitope breadth.

### 6.2. Polyvalent Mosaic mRNA Vaccine for PRRSV

PRRSV infections mainly cause respiratory disease in piglets and reproductive failure in sows, resulting in great economic losses in the swine industry. PRRSV strains were classified into two distinct genotypes with distinct antigenicity. Vaccinations with live attenuated or inactivated PRRSV vaccines have played a significant role in controlling PRRSV spread but with significant drawbacks.

Live attenuated PRRSV vaccines showed high efficacy against genetically homologous PRRSV strains, but only conferred partial or no protection against heterologous strains [118]. In addition, virulent circulating vaccine-derived PRRSV strains were identified from the swine farms in both China and the United States, where the live attenuated vaccines are widely used, raising concerns about the safety of current attenuated vaccines [119,120]. Compared with the live attenuated vaccines, inactivated PRRSV vaccines are much safer, but are less effective against virus infection in the vaccinated herds.

Thus, there is an urgent need for a safe and effective vaccine to control PRRSV. Given the high antigenic and genetic diversity of PRRSV, the mosaic vaccine approach using mRNA technology might offer one potential solution. In 2019, a DNA vaccine candidate containing PRRSV GP5-Mosaic sequences was shown to be immunogenic and induced protection of pigs against virus infection, supporting the potential for the mosaic vaccine to provide broader protection against emerging PRRSV variants [121,122]. The fragments in PRRSV encoding regions that potently elicit immune responses could be selected from multiple circulating strains via in silico algorithms and then assembled into a single vaccine using the mRNA vaccine platform. Such vaccines encoding synthetic mosaic antigens should induce more diverse and effective immune responses with greater variant depth.

### 6.3. RBD-Based mRNA Vaccine for Animal Coronavirus

Coronaviruses infect a wide range of animal species, including pigs, cows, cats, and dogs. They have caused several emerging pandemics, including severe acute respiratory syndrome (SARS) in 2003, Middle East respiratory syndrome (MERS) in 2012, and the novel coronavirus disease in 2019 (COVID-19) [123]. More importantly, certain coronaviruses can spill over from the reservoirs to humans via potential intermediate host species, threatening public health [124]. Coronaviruses in different animal species share genetic structure similarity and encode spike (S) glycoprotein, which is the main target of neutralizing antibodies. The S protein is composed of the S1 and S2 subunits. The S1 subunit contains a receptor-binding domain (RBD) that is responsible for specific receptor binding, while the S2 subunit mediates the fusion between viral membrane and host cell membrane. Importantly, various lines of evidence demonstrated that the RBD is a major target of neutralizing antibodies; thus, RBD is one of the prominent targets for vaccine development.

As the COVID-19 pandemic occurs, the RBD-based mRNA vaccines have been developed by multiple groups. The safety and immunogenicity data from the clinical trials supported that an RBD-based vaccine candidate (BNT162b1) was well tolerated and immunogenic. Compared with the mRNA vaccine (BNT162b2) encoding a membrane-anchored SARS-CoV-2 full-length S protein that is stabilized in the prefusion conformation, the BNT162b1 elicited similar dose-dependent neutralizing antibodies in both younger and older adults, despite a higher incidence and severity of systemic reactions, particularly in older adults [125,126]. Moreover, Moderna’s mRNA-1273 vaccine-elicited antibodies were more targeted to the RBD and showed greater binding breadth and efficacy, compared to antibodies elicited by natural infection [127]. Therefore, RBD is a major target for coronavirus vaccine development.

In terms of animal coronaviruses such as PEDV, bovine coronavirus (BCoV), transmissible gastroenteritis virus (TGEV), and the recently emerged swine acute diarrhea syndrome coronavirus (SADS-CoV), only limited vaccines are currently available, and most of the licensed vaccines are inactivated or live attenuated. With the advances in understanding of the RBD and cellular surface receptors of animal coronaviruses, RBD-based mRNA vaccines seem to be a very promising strategy for combating the emerging and re-emerging animal coronaviruses.

### 6.4. E2-Based mRNA Vaccine for Pestiviruses

Pestiviruses within the Flavividae comprise three economically important animal viruses, including BVDV, border disease virus (BDV), and classical swine fever virus (CSFV). Pestiviruses encode three glycoproteins, E^rns^, E1, and E2, among which the E2 protein is the major target for neutralizing antibodies. Previous findings demonstrated that the recombinant E2 subunit vaccines of BVDV and CSFV could completely provide protection against virus infection. Both humoral and cellular-mediated immunity play important roles in clearing CSFV and BVDV infection upon vaccination [128,129]. Thus, immunization with the E2-based mRNA vaccines might be more effective due to the activated humoral- and cellular- mediated immune responses.

As noted above, ZIKV prM-E-mRNA vaccine elicited not only protective antibody response, but also E protein-specific CD4^+^ and CD8^+^ T cell responses [66]. Thus, mRNA vaccines are promising alternatives to traditional vaccine approaches for preventing the spread of pestiviruses due to their high efficacy.

In conclusion, mRNA vaccines have great potential in the veterinary field against animal infectious diseases, particularly the zoonoses. The advantages include no risk of infection or reversion to virulence, simultaneous immunizations against multiple pathogens, and relatively ease of design, enabling the RNA vaccine platform to overcome the bottlenecks faced by the traditional platform. Nonetheless, setbacks such as the instability of the vaccines, the relatively high expense of manufacturing them, and current delivery limitations impede the use of mRNA vaccines in the veterinary field. Currently, intramuscular, intradermal, or subcutaneous injection are the main delivery routes for mRNA vaccines in human. To reduce the stress, pain and cost of vaccinations, noninvasive delivery routes, including oral delivery and aerosol administrations, are becoming the preferred routes for animal vaccines. Thus, the design of new carriers should help improve the efficacy and stability of mRNA vaccines for the noninvasive delivery routes. Furthermore, adverse events and allergic reactions from the delivery of mRNA vaccines need to be evaluated in the natural animal hosts. More importantly, animal vaccines must be affordable. However, the cost of mRNA vaccines remains high due to the use of high-priced reagents and the cold chain infrastructure. Given the fast pace of progress in mRNA vaccines, especially in the areas of immune-informatics and in vivo delivery methods, the future of mRNA vaccines at a low cost and high quality in the veterinary field is extremely bright.

## Figures and Tables

**Table 1:** Summary of advantages/disadvantages characteristics of main types of vaccines.

	Types	Inactivated Vaccines	Live Attenuated Vaccines	Recombinant Subunit Vaccines or Synthetic Peptide Vaccines	Recombinant Live Vectored Vaccines	DNA Vaccines	mRNA Vaccines
Characteristics							
**Advantages**	Strong humoral immune responses	+	++	+	++	+	+
	Strong cellular immune responses	-	++	-	++	+	++
	Rapid development and production	+	-	+	+	++	++
	Discrimination between infected and vaccinated animals	+	-	++	++	++	++
	Good stability	+	+	+	+	++	-
	Convenient storage conditions	+	-	+	-	++	-
**Disadvantages**	Infectious	N	Y	N	Y	N	N
	Reversion or mutation	-	++	-	+	-	-
	Risk at genetic integration	-	+	-	+	++	-
	Adjuvants required	Y	N	Y	N	N	N

“++, +, -”: high score to low score; “Y”: Yes; “N”: No.
